# Severe peripheral blood lymphopenia without NK cell cytotoxicty deficiency is the rule in adult acquired HLH

**DOI:** 10.1186/1546-0096-13-S1-O26

**Published:** 2015-09-28

**Authors:** J Carvelli, C Piperoglou, F Vely, C Farnarier, K Mazodier, J-R Harle, E Vivier, G Kaplanski

**Affiliations:** 1 CHU Conception, Service de Médecine Interne et Immunologie Clinique, Marseille, France; 2 CHU Conception, Laboratoire d'immunologie, Marseille, France

## Introduction

Hemophagocytic lymphohistiocytosis (HLH) is characterized by hypercytokinemia and hemophagocytosis due to abnormal activation and proliferation of T lymphocytes and macrophages. Inherited forms are due to gene defects affecting CD8 and NK lymphocyte cytotoxicity, whereas in acquired forms complicating rheumatic, infectious or neoplastic diseases, lymphocyte subpopulation and function profiles remain poorly studied.

## Methods

Between 2000 and 2014, we prospectively studied 71 adult patients (from 20 to 87 years) with acquired HLH defined by the International HLH Society criteria (group 1), compared with 87 patients suffering of rheumatic, infectious or tumoral diseases without HLH (group 2) and 66 healthy volunteers (group 3). Peripheral blood lymphocyte phenotype was studied by FACS analysis with special focus on NK cell activation markers and functions.

## Results

Acquired HLH was mainly associated with infections (n = 33), lymphomas (n = 14), inflammatory pathologies (4 lupus, 2 others connective tissue diseases, 3 adult onset Still's diseases) or undetermined causes (n = 15). HLH patients had a global lymphopenia compared to group 2 and 3 affecting both T, B and NK cells (p < 0.0001) which was transient and corrected in case of HLH recovery. Among CD3 cells, both CD4 (mean: 333 vs 638/mm^3^) and CD8 (272 vs 638/mm^3^) were lower in HLH compared to group 2 (p <0.0001). NK cell phenotype showed membrane CD69 and CCR5 up-regulation reflecting NK cell activation, but no differences for NKp30, p46, CD16, CXCRC1, CXCR3 and CCR7 membrane expression. NK cytotoxic functions consisting in perforin expression, LAMP degranulation ability, NK natural cytotoxicity or ADCC against K562 or P815 targets respectively, appeared not to be affected in acquired HLH and completely different from the severe functional defects despite normal cell counts, observed in patients with inherited HLH.

## Conclusion

Compared to inherited forms of HLH and matched patients with rheumatic, infectious or neoplastic diseases, patients with acquired HLH present with a severe but transient global lymphopenia. Despite severe decreased NK cell counts, these cells appeared to express several activation markers and overall a normal phenotype. More importantly, NK cells demonstrated normal cytotoxic functions. Despite some reports showing mutations affecting T cell cytotoxicity genes in acquired HLH patients, our study shows that this disease is in most of the cases, not due to a defect of lymphocyte cytotoxicity.

